# Lectures on Clinical Surgery

**Published:** 1855-08

**Authors:** James Syme

**Affiliations:** Professor of Clinical Surgery in the University of Edinburg


Lectures on Clinical Surgery. By James Syme, Esq., Professor of Clinical Surgery in the University of Edinburg.

Lecture XVII. *Lateral Curvature of the Spine.*—Hitherto, gentlemen, the remarks which I have had the pleasure and honor of addressing to you have always had reference to particular cases; but I propose to-day to offer you some observations not in connection with a case. My reason for this deviation from the usual practice is, that the derangement which I wish to bring under your consideration never can be treated under my care in this hospital, while at the same time the subject is of great importance, and one regarding which I am anxious that you should entertain just ideas: I allude to Lateral Curvature of the Spine. I suppose I need not remind you that the vertebral column is made up of a number of bones, with interposed plates of fibro-cartilage and connecting ligaments, and that muscles are attached which give it stability and also execute its movements. The spine thus constitued, when viewed from before backwards, appears nearly straight in the healthy condition, having merely a slight lateral curve in the dorsal region; while seen laterally it presents a series of curves, having a convexity forwards in the cervical and lumbar, and a concavity in the dorsal region, and is thus much better adapted for the erect posture than if the dorsal and lumbar portions formed but one curve, as is the case with quadrupeds, and even the quadrumana. The spine tends to yield in advanced age, when its natural curves become increased, and the trunk is proportionately shortened, and indeed in our diurnal experience the same thing occurs to some extent, so that a man is taller in the morning than after the fatigue of the day—a fact of which recruiting sergeants are well aware, and when, therefore, there is any question as to stature, they take care to have it ascertained in the early part of the day. But such changes of form in the spine are of slight consequence compared with the great morbid deviation to which it is liable in the lateral direction. This occurs chiefly in the dorsal region, where a curve is formed with its convexity generally to the right, though sometimes to the left, while in the lumbar region a compensating curve occurs in the opposite direction. This distortion may exist to any extent, from that which is so slight as to require the greatest nicety to discover it, to the extreme degree to which you see in this skeleton. You observe the bodies of the vertebræ are of a wedge shape where the curvature exists, and they have also undergone rotation, so that their anterior part constitutes the convexity of the curve; the ribs have also suffered, being on the right side separated one from another, and broader than natural, their angles being so acute, that some of them are actually in contact with the bodies of the vertebræ, while on the left side they are pressed together and very narrow. You will readily understand how this state of things, besides the great deformity which it produces, must greatly cramp the organs contained within the chest, and interfere with their functions. But leaving the effect of this disease, I must pass to what I wish to bring particularly under your notice at present—its causes, prevention, and remedy.

In considering the causes, you must remember, that in order to keep the spine erect, a muscular effort is required; if you attempt to place in the erect posture the body of a drunken, sleeping, or dead man, the trunk will bend from the want of this action. If the exertion be long-continued, the muscles get tired, more especially if the individual be not strong, and he seeks relief from support, if possible, but in the absence of it, allows the spine to bend so far as the resistence of its textures admits, and this generally takes place in the lateral direction. If the position thus assumed be long maintained, and frequently repeated, it is apt to become permanent; the bones yielding and becoming altered in form to accommodate themselves to the unnatural position, and when once this change has commenced in the bones, it goes on more and more rapidly, the weight of the head and upper part of the body acting with greater leverage, and therefore, with more effect. Lateral curvature does not generally occur without a predisposition, and among the predisposing causes, age is one of the most important. It may be said to be essentially a disease of youth, occurring generally between seven and seventeen, and most frequently about the middle of this period. Females are more subject to it than males, and especially those of weak constitution, and who have been delicately brought up. There is also, sometimes, a predisposition from an unnaturally soft state of the bones, and independently of these causes, one often sees evidences of an hereditary tendency to the disease, from its appearing in several members of the same family, sometimes in short squat persons, whom you would think not likely to be affected with it. While the disease is thus generally induced by fatigue of the muscles, in consequence of long continuance in one position, it may occur in the opposite condition—namely, from inordinate muscular efforts. If you wish a muscle to act so as to produce an effect upon its insertion, it is necessary that its origin be fixed, for which purpose another set of muscles are called into action. In this way a vigorous effort of the right hand brings into play the muscles of the trunk, and the chest is bent to the opposite side. Hence the cause why hernia is so common on the right side, the right hand being more frequently used than the left, and when the chest is thrown over to the left side, the pressure of the diaphragm tends to force the abdominal viscera more to the right than to the left groin. Excessive muscular exertion is, however, a much less frequent cause of curvature than the opposite condition of deficient exercise in the erect or sitting posture. Tight stays, by interfering with the action of the muscles of the trunk, may have an indirect effect in producing lateral curvature. Much nonsense has been spoken about the influence of stays in this disease, but they cannot act except in this way.

With regard to the means of treatment that are in use, the first I have to mention to you consists of exercise of the muscles of the trunk, it being supposed that as want of exercise is a great cause of the disease, exercise must be the best means for its remedy. Nothing can be more absurd than this practice; the spine is already curved, and its bones altered in form, and you cannot correct this condition by exercise; on the contrary, the more the patient is kept in the erect posture, the greater tendency will there be to increase of the deformity. Exercise might have prevented the disease, but if employed subsequently to its establishment, must increase the evil. I recollect seeing a young lady from a boarding-school in England, who had been sent out on botanical excursions several hours every day, on account of lateral curvature, with no better result than rendering the deformity extreme. This notion of affording relief by exercise, has been cherished to a degree of absurdity that you could hardly conceive possible. It has been supposed that because milkmaids and others who carry heavy weights upon their heads, are commonly erect in their gait, it must, therefore, be useful for persons affected with lateral curvature, to sustain similar loads, and accordingly, footstools filled with books, etc., have been placed upon the heads of young ladies laboring under this distortion. I am happy to be able to quote on the subject of exercise as a means of treatment, an authority which you will be better able to appreciate bye-and-bye—namely, the surgeon of the Royal Orthopœdic Hospital. He writes: "Exercises are of use only where we find nothing more than the disposition to spinal curvature. In delicate patients, well-regulated and gentle exercise, with good diet, cold bathing, bark, and chalybeates, will effect wonders in the way of prevention; but when a cure is called for, other means must be put into operation. No one will deny that the exercise of muscles increases their development. Therefore, to exercise the muscles of a curved back by the use of pulleys, and a variety of other modes, is to create a direct obstacle to successful treatment, for not only are the muscles exercised on the convexity of the curve, but also on the concavity, which, by an increase of development, augments the resistance already present,"* and in illustration of the system thus more decidedly than lucidly impugned, Mr. Tamplin gives the following "as a literal statement made to him by a patient from a spinal hospital, of the course of treatment she received there; nine o'clock till one, swinging, climbing a ladder, and turning a wheel; then hung up by the head for half an hour; then laid on a couch for an hour; then dinner; laid on the couch till five; then a walk (in summer); then to bed."† This quotation will serve to show you, that independent of any argument founded upon a common-sense view of the subject, we may demolish, by orthopœdic authority, the treatment by muscular exercise. But I must warn you, that you will find the friends of patients very partial to this kind of treatment. I recollect being called to see a young lady with lateral curvature, whose father was a great philosopher. In her boudoir, which might well be called a chamber of horrors, was a long ladder, a swing suspended from the ceiling, a wheel for her to turn, and various other implements of torture lying about the floor. I tried to explain to this gentleman that as the curvature was already present, all that exertion could be productive of no benefit; but he was not to be convinced, and I dare say that young lady is to this day climbing her ladder, turning her wheel, and going through the rest of her gymnastics.

Passing from the treatment by muscular exercise, there are two other means only to be considered. Both of these are founded on mechanical support, which is applied on two principles; one of which is to open up the curve by pressure applied to its extremities; the other to force the distorted spine into its proper position by pressure on the convexity of the curve. We will consider these methods in order. The apparatus by which the former object is alleged to be obtained, consists of an iron hoop round the pelvis, from which a crutch passes up at each side to support the arm-pits. Just consider for a moment how this will act. The scapulæ being connected with the spine only by muscles, pressure applied below the arms may raise them (the scapulæ), but cannot alter the position of the spine, and its only effect will be to take off the weight of the upper limbs, which is comparatively slight, while that of the head and upper part of the trunk continues to operate as injuriously as ever. There will, no doubt, be an improvement in the patient's figure as long as the support is applied, but no change so far as the spine is concerned. This is so obvious

---

* Vide Lateral Curvature of the Spine, by R. W. Tamplin, p. 34.

† Vide op. cit., p. 36.

that I need hardly detain you longer upon it; and, accordingly, I find that the plan now generally used is different, and that the principle of opening up the curve, which was pursued at a former period, is now generally superceded by the other principle of compressing the convexity, the apparatus being so constructed that plates of metal, or bands of some firm material, are forcibly applied over the prominence of the distorted ribs. If you look at this preparation, you will see that pressure so directed cannot possibly have any good effect, but must aggravate the complaint. You may say this is prejudice on my part; but I will again bring you an unimpeachable authority, and as I before quoted the surgeon of the Royal Orthopœdic Hospital, I will now quote the assistant-surgeon of the institution: "There are many forms of portable instruments," writes Mr. Brodhurst, "though all are made on one or two principles of action. Thus, one kind acts on the convexity of the curve; the other, on the superior extremity of the primary curve. Of the former is the instrument invented by Delpech, and used by Stromeyer, and at present by Mr. Tamplin; and also that of Tavernier, and its modification known as Mr. Lonsdale's. The former consists essentially of metal plate, which presses on the convexity, and, laterally, on the ribs; the latter, of a webbing band, which presses posteriorly and laterally. Many others, somewhat differing in form, act in the same manner."* Now, I beg you will attend to the following statement regarding these apparatus by the assistant surgeon of the Royal Orthopœdic Hospital: "Every instrument which acts on the spine by pressure laterally on the ribs, must, after rotation has taken place, increase the flattening of the ribs; and must also increase, through pressure on the convexity, the wedge-shape of the bodies of the vertebræ. Much might be said on this subject. I will, however, only further introduce a few lines from the late Mr. Shaw's work: 'There can be little doubt,' wrote Mr. Shaw, 'that a lateral pressure made upon the ribs, when the spine is slightly bent, will tend to throw the sternum forwards, and thus to give the chest not only the form resembling that of a bird, but even of a fish. And, again, it is easy to demonstrate that the application of an instrument, with this intent (lateral pressure), not only diminishes the area of the chest, but increases the distortion; the shoulder is pressed against the flat part of the ribs, and, consequently, the unnatural angle, which even in cases of slight distortion is, to a certain degree, formed in the ribs, is increased. When the ribs are thus distorted, the size of the chest is very much diminished, for the middle of the rib is necessarily brought nearer to the spine.'"† Now, if any one not a specialist, were to express such

---

* Vide on Lateral Curvature of the Spine, by Bernard E. Brodhurst. 1855. p. 51.
† Vide op. cit. p. 53.

sentiments, he might be met with the retort that he was an ignoramus, and therefore not to be listened to; but no such objection can be made here, and I confess I am not sorry to see these signs of insubordination approaching to mutiny in the orthopœdic camp; and I can but hope that the quarrel thus begun will be carried on with acrimony. It is a common saying, that if a certain description of people quarrel, the rest of the community derive benefit; and I should not be sorry if the conflict in this case should be carried no less bitterly than the famous encounter of the Kilkenny cats, which, you may recollect, devoured each other so effectually that nothing remianed ot them but their tails.

I hope I have said enough to satisfy you that muscular exercise and mechanical support, on whichever principle it be applied, are alike ineffectual in the treatment of the disease. Are we, then, to regard lateral curvature as incurable? I be.ieve we must admit that it is so if the bones are altered in shape. How is it, then, you may ask, that we find so many cures related, and that the orthopœdic system is applied so extensively to the disease, even in its most advanced stages? This is not difficult to understand. A young lady from the country, affected with spinal curvature, consults an orthopœdist, who prescribes steel stays, which have the effect of improving her figure while her clothes are on, and letters are written to her friends at home, reporting the wonderful improvement. After a while it is found that when the stays are taken off things are much as they were at first; this is attributed to some imperfection of the apparatus, and alterations are made in it with no better result; and this goes on and on till the patient's friends are tired; but in the meantime the first effect of the favorable news has become extensively diffused, while the final failure is not talked of. Why should it be? The disappointment, and loss of time as well as money, are not pleasant subjects of conversation. Hence the effect of the flourish of trumpets that is sounded on the first supposed improvement is not counteracted by the ultimate result. Another reason why cures are ascribed to this treatment is, that the stays are often applied to where no curvature exists at all. This very apparatus, heavy, complicated, and ingeniously constructed as you now see it, I directed to be removed from a young lady, whose spine was and, so far as I could learn, always had been perfectly straight. You will inquire why such things are done. A panic exists among the public on the subject of spinal curvature, and you will find that in almost every boarding-school for young ladies, there lies on the drawing room table, one or more neatly-bound and strikingly-illustrated works on curvature of the spine. Mr. Tamplin quotes a description, the accuracy of which he "can personally vouch from repeated inquiries," and in it the following statements occur: "We lately visited, in a large town, a boardingschool containing forty girls; and we learned, on close and accurate inquiry, that there was not one of the girls who had been at the school two years (and the majority had been as long), that was not more or less crooked." "We can assert, on the same authority of personal observation, and on an extensive scale, that scarcely a single girl (more especially of the middle classes) that has been at a boarding-school for two or three years returns home with unimpaired health."* Well, then, you can fancy what a fine field there is for this practice. If any young lady is observed to sit a little awry, a spine doctor is called in, and stays are ordered for her. I do not say that the wearing such apparatus necessarily induces curvature, though I do not doubt it must fatigue and encumber the patient so as to increase the tendency to it; and supposing that the spine even should remain straight when the stays have been worn for a length of time, the case is considered one of cure by the apparatus. I could mention to you many remarkable cases that have come under my notice, where steel stays have been long worn, while nothing was wrong in the spine.

The confidence too generally placed in this mode of treatment arises, then, from two causes—namely, the natural sanguine expectations of the patients' friends, excited by the machine-makers, and not counteracted in their effects by the final failure; and, secondly, from the apparatus being often applied where no disease is present. I may remark, that what I am stating to you is nothing new—nothing more than was well-known five-and-twenty years ago, when the futility of mechanical support had been clearly demonstrated. But a strange revolution has since taken place. When the orthopœdic system commenced, it was alleged that lateral curvature of the spine could be cured by cutting the muscles of the back. Nothing could be more monstrous than such a proposal, and I then felt it my duty to write a paper, in pretty strong terms, upon the subject. It would now be needless to say anything against this practice, for its absurdity is admitted on all hands; but at that time it was taken up extensively by the public, and cases of lateral curvature thus came to be claimed by the orthopœdists, who, having got possession, kept a firm hold, and now, dismissing all pretence to tenotomy, subject them to mechanism, far beyond any extent ever previously practiced. It is therefore the more necessary that we who practice the profession in a comprehensive manner should tell the plain truth to our patients upon this subject; but while confessing that when the disease has advanced to alteration of form in the bones, cure is beyond our power, you should throw your strength into the statement of the important truth that where only a tendency to the disease is present, it can be easily and effectually obviated by

---

* Vide Tamplin on Lateral Curvature, p. 9.

proper management. If a young lady shows her disposition to latetal curvature, you may certainly prevent its occurrence by enjoining upon her friends not to allow her either to sit still or to stand still—a precept which should be written in letters of gold—but in the intervals of the periods allowed for varied and active exercise to recline upon a mattress or sofa when she reads or works. Reclining-boards are absurd contrivances, which should be discarded. I recollect a lady who came from the country with her daughter, having previously written about her case, and who said that she was ashamed to bring her, as there was then nothing at all the matter with her back, but that as she had arranged to come she thought it best to do so. It appeared that in the interval from the time when she wrote, the young lady had been relieved from her lessons, and was soon afterwards observed to be free from all appearance of deformity. But when the alteration of form has occurred in the bones, the next best service you can render is to keep your patients out of the hands of those rapacious individuals who, while they hold out delusive assurances of relief, extort money which can often be ill afforded, and lead in the end to nothing better than disappointment. If consulted when the patient is not grown up, and the bones are but slightly affected, you may do much good by enforcing the recumbent posture, when the future growth of the bones may, and probably will, prevent aggravation of the complaint; even here, however, a cure of deformity already existing must not be looked for.

I will trouble you with no more observations on this subject at present, trusting that what has been said will induce you to give it your serious consideration, and study it as you would any other department of surgery, without being trammelled by popular prejudice or the ignorant dogmatism of specialists.—*London Lancet.*

